# Resonant Dyakonov–Shur Magnetoplasmons in Graphene Terahertz Photodetectors

**DOI:** 10.1002/nap2.70094

**Published:** 2026-04-21

**Authors:** Juan A. Delgado‐Notario, Cedric Bray, Elsa Pérez‐Martín, Benjamin Benhamou‐Bui, Namrata Saha, Sahil Parvez, Christophe Consejo, Guillaume Sigu, Salah Benlemqwanssa, Laurent Bonnet, Takashi Taniguchi, Kenji Watanabe, José M. Caridad, Sergey S. Krishtopenko, Yahya M. Meziani, Benoit Jouault, Jérémie Torres, Sandra Ruffenach, Frédéric Teppe

**Affiliations:** ^1^ Departamento de Física Aplicada Universidad de Salamanca Salamanca Spain; ^2^ Unidad de Excelencia en Luz y Materia Estructurada (LUMES) Universidad de Salamanca Salamanca Spain; ^3^ L2C (UMR 5221) Université de Montpellier CNRS Montpellier France; ^4^ Research Center for Materials Nanoarchitectonics National Institute for Materials Science Tsukuba Japan; ^5^ Research Center for Electronic and Optical Materials National Institute for Materials Science Tsukuba Japan

**Keywords:** detector, graphene, magnetoplasmons, TeraFET, THz, transistor

## Abstract

Graphene plasmons confine incident terahertz fields far below the diffraction limit and, when hosted by a gate‐defined Fabry–Perot cavity, they enable electrically tunable, frequency‐selective photodetectors. In a magnetic field, these plasmons hybridize with the cyclotron motion to form magnetoplasmons, offering a platform for fundamental studies and for nonreciprocal, spectrally selective, and ultrasensitive terahertz photonics. However, implementing magnetoplasmon‐assisted resonant transistors at terahertz frequencies has remained challenging so far. Here, we extend the resonant Dyakonov–Shur graphene TeraFET framework into the magnetoplasmonic regime and use gate‐dependent, on‐chip terahertz photocurrent spectroscopy combined with a perpendicular magnetic field to resolve and probe the evolution of resonant magnetoplasmons in antenna‐coupled monolayer and bilayer graphene TeraFETs. In monolayer graphene, the dispersion reflects the Dirac nature of the carriers, exhibiting a nonmonotonic density dependence due to the interplay of plasma resonance and cyclotron motion, with an inflection point at maximal plasmon–cyclotron coupling. In contrast, in bilayer graphene, we recover and map a magnetoplasmon dispersion consistent with the conventional Schrödinger‐type picture. These results establish graphene TeraFET devices as a robust on‐chip platform for resonant magnetoplasmonics at terahertz frequencies, opening avenues toward magnetically programmable, frequency‐selective terahertz photodetectors.

## Graphical abstract

Cavity‐confined Dyakonov–Shur plasmons in graphene evolve into magnetoplasmons by an external perpendicular magnetic field. Gate‐tunable THz photocurrent spectroscopy reveals their dispersion over a broad frequency range, uncovering a nonmonotonic behavior in monolayer graphene in contrast to the conventional response of bilayer graphene.

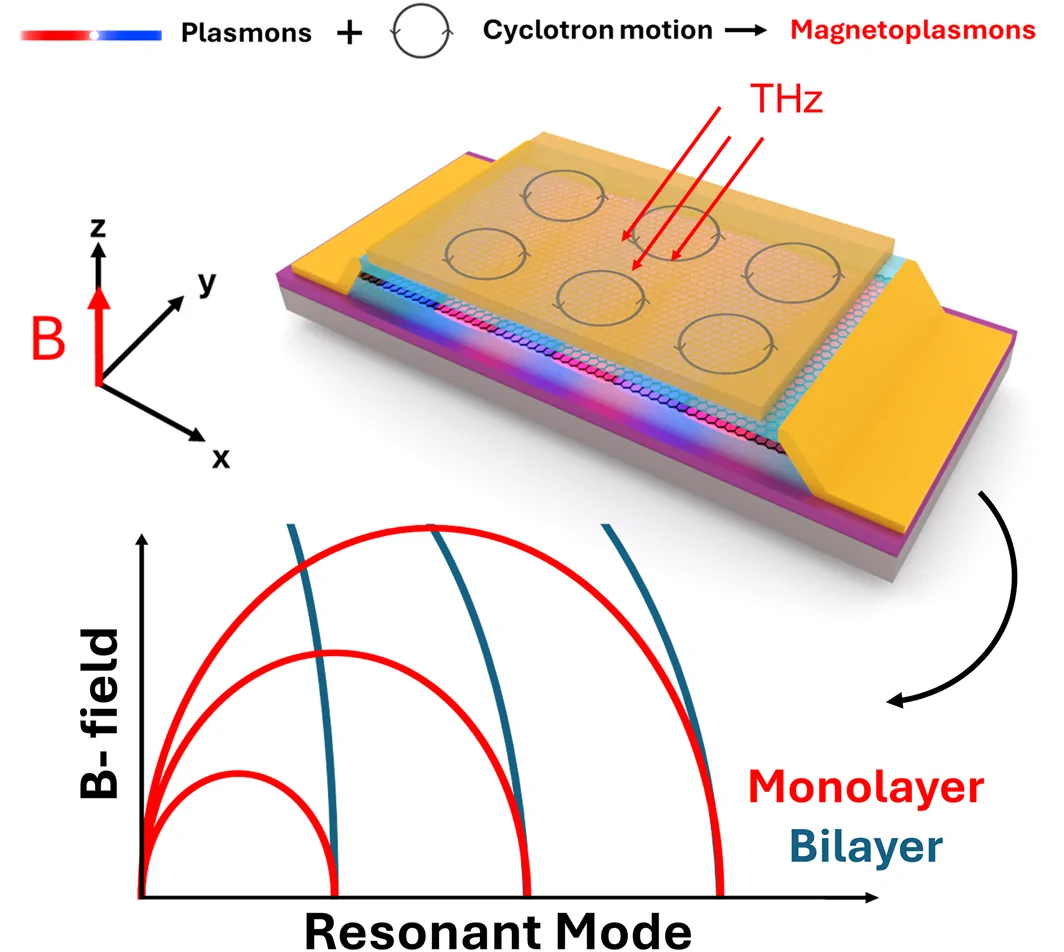

## 1 Introduction

The ability to control light–matter interactions at the nanoscale in the terahertz (THz) range has attracted significant attention at the interface of fundamental research and device technology [1]. A particularly powerful pathway is offered by plasmons—coherent charge density oscillations on a conductor/dielectric interface that compress incident fields deep below the diffraction limit and propagate along the surface—promising transformative advances in the THz‐band for applications in the fields of spectroscopy, emission, sensing, bioimaging, chemical detection, and ultra‐broadband wireless communications [2, 3, 4, 5, 6]. A groundbreaking route to harness these light–matter interactions in electronic platforms was theoretically proposed 3 decades ago by M. Dyakonov and M. Shur [7, 8], suggesting that field‐effect transistors operating at THz frequencies (TeraFETs) can exhibit resonant response to the incident electromagnetic radiation at the eigenfrequencies of plasmonic oscillations of the two‐dimensional electron gas (2DEG) in the device's channel. In these devices, the gated channel creates a tunable plasmonic cavity whose discrete modes are set by device length, carrier density, and boundary conditions. This challenging regime, the so‐called resonant operation scenario, is characterized by a quality factor *Q = ωτ ≫* 1 (where *ω =* 2π*f* is the angular frequency of the incident radiation and *τ* the momentum relaxation time of the charge carriers in the system). Such conditions are rarely met in most electronic systems, in sharp contrast to the more common overdamped regime *Q ≪ 1*, where plasmons are heavily damped in the channel and decay before even reaching the other side of the plasmonic cavity. Since this theoretical proposal, many materials and device architectures have been explored to exploit plasmons in TeraFETs, predominantly in the overdamped (nonresonant) regime, which results in broadband rectification [9, 10, 11, 12, 13, 14, 15, 16, 17]. By contrast, the resonant regime has proven much harder to access, with only a limited number of demonstrations, including submicron III–V transistors and plasmonic cavity devices [18, 19, 20, 21, 22, 23, 24]. In this context, graphene TeraFETs have shown renewed interest in this elusive field by demonstrating its gate‐tunable resonant THz detection under zero source–drain bias, including multiple harmonic plasma resonances in bilayer graphene [25] and resonant signatures persisting up to room temperature in single‐layer graphene [26].

Despite substantial progress in recent years, research on plasma‐wave–assisted resonant operation in graphene‐based TeraFETs has, to date, largely focused on tuning plasmons via excitation frequency, channel length, temperature, and carrier density [25, 26], whereas the effect of a perpendicular magnetic field on cavity‐confined Dyakonov–Shur plasmons has remained essentially unexplored. Interestingly, when subjected to a perpendicular magnetic field, plasmons, irrespective of the host material or device architecture, can couple with cyclotron resonance to form hybrid modes known as magnetoplasmons. These modes result from the interplay between collective charge oscillations and the cyclotron motion of charge carriers and offer unique opportunities for investigating material properties as their dispersion encodes information on carrier effective mass, electronic interactions, and hydrodynamic effects in electron liquids [27, 28], enabling appealing THz photonic applications, such as nonreciprocal plasmonic platforms [29] and ultrasensitive label‐free biosensors [30]. So far, the existence of graphene magnetoplasmons and their dynamics have been studied across a variety of device geometries and/or experimental approaches. For instance, THz time‐domain spectroscopy (THz‐TDS) and FTIR transmission measurements have revealed the presence of magnetoplasmons in epitaxial graphene structures and nanoribbons [31, 32], highlighting the impact of impurities and confinement effects on plasmon frequencies [28]. Furthermore, studies have highlighted the existence of magnetoplasmonic modes in graphene structures, revealing distinct edge and bulk(‐like) modes [33, 34], and more recently, Bernstein magnetoplasmon resonances near cyclotron harmonics [35]. Also, by incorporating periodic nanostructures of disks, nanoribbons, or split rings, it becomes possible to engineer active metasurfaces capable of dynamically modulating the THz transmission, absorption, and/or polarization spectral features, while also enabling the study of the optical nonlinearities of graphene magnetoplasmons [5, 36, 37]. Nevertheless, these studies fall outside the Dyakonov–Shur resonant plasma‐wave framework, leaving the role of the magnetic field on cavity‐confined graphene THz plasmons largely unexplored.

In this work, we combine THz photocurrent spectroscopy (discrete frequencies between 0.25 and 3.8 THz) with gate‐controlled carrier density and an external perpendicular magnetic field to probe cavity‐confined resonant magnetoplasmons in graphene TeraFETs under the presence of magnetic fields and demonstrate the coupling between Dyakonov–Shur standing plasma waves in the gated channel region and the cyclotron resonance. To do that, we used high‐quality mono‐ and bilayer graphene encapsulated between hexagonal boron nitride (hBN) flakes to provide the clean environment necessary to excite and support long‐life graphene plasmons as demonstrated in recent works [25, 26]. Using single top‐gated antenna‐coupled TeraFETs, THz photocurrent spectroscopy technique enables the systematic study of magnetoplasma resonance modes as a function of frequency, carrier density, and magnetic field, for both massless Dirac fermions in monolayer graphene and massive Dirac fermions with parabolic dispersion in bilayer graphene. This study provides deeper insight into the behavior of magnetoplasmons in graphene, particularly in the Dyakonov–Shur regime of standing plasma waves confined in the plasmonic cavity under the gate. In addition, our results pave the way for future investigations involving magnetoplasma waves in more complex device architectures, with potential applications in magnetoplasmonic interferometry [38, 39], exploring the hydrodynamic properties of 2D Dirac plasmons [40] under a magnetic field and magnetically tunable, frequency‐selective photodetectors [41].

## 2 Methods

To qualitatively probe the behavior of resonant graphene magnetoplasmons subjected to an incident THz radiation in the presence of a magnetic field (See Figure 1a), our graphene TeraFET devices studied in this work were fabricated from mechanical exfoliated single‐layer (Dirac‐type) and bilayer (Schrödinger‐type) graphene encapsulated in between thin hBN flakes using a van der Waals dry‐assembling technique. All the graphene‐based TeraFETs in this study were defined to have a short channel (*L*_ch_ = 6 μm) with side contacts for the drain and source electrodes. A metal top‐gate electrode covering most of the TeraFET channel (*L*_TG_ = 4.8 μm) was subsequently patterned to create the plasmonic cavity. To couple the incident electromagnetic waves and enable the excitation of resonant THz standing plasma waves below the gated section of the channel, a coupled bow tie antenna was integrated between the top gate and the source electrode (See Figure 1b). More information about the fabrication process can be found in Supporting Information S1: Note 1.

**FIGURE 1:**
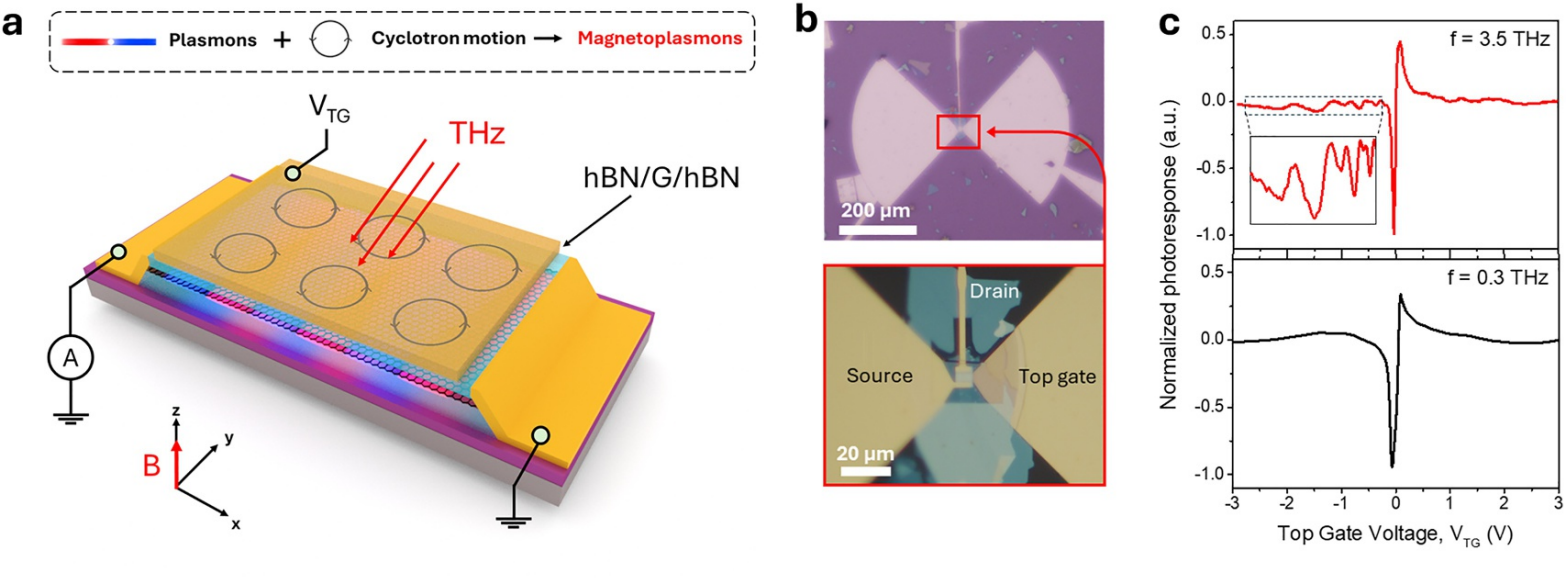
Graphene‐based TeraFET photodetector. (a) Schematic 3D view of the THz photocurrent measurements of the device in the presence of a magnetic field highlighting the presence of resonant graphene magnetoplasmons under the gate (The cavity‐confined plasmon waves are schematically shown in blue and red colors, whereas the cyclotron motion is represented by circular orbits). (b) Optical microscope image of the single‐layer graphene THz detector, featuring a bow tie antenna integrated between the source and top‐gate electrodes. The bottom panel provides a zoomed‐in view of the device, with source, top gate, and drain contacts labeled. (c) Nonresonant (bottom) and resonant (top) normalized THz photoresponse as a function of the top gate voltage, V_TG_, measured for the monolayer TeraFET at 0.3 and 3.5 THz, respectively, for a temperature of 1.7 K and a zero magnetic field.

The devices were placed inside a cryogenic magnet system (up to 6 T) with optical access and cooled down to a temperature of 1.7 K. All results presented in this work were obtained at this operation temperature unless otherwise indicated. THz photoresponse measurements (Figure 1a) were performed at zero source–drain bias at different carrier densities, under a wide range of THz excitation wavelengths and/or in the presence of magnetic fields ranging up to 1.5 T (Here, the magnetic field was oriented perpendicular to the surface of the sample and parallel to the wave vector of the incident radiation). Carrier density was tuned in our TeraFETs using a voltage generator to control the applied voltage on the top‐gate electrode. In order to study the photoresponse of our detectors at different frequencies, we used two radiation sources. Low THz measurements were carried out using a Schottky diode‐based frequency multiplier source generating tunable THz wavelengths from 0.220 to 0.330 THz with average output power around 10 mW. For higher THz frequencies, we used a quantum cascade laser to illuminate the sample at fixed frequencies of 2.5, 2.9, 3.3, 3.5, and 3.8 THz (optical powers are about 2.5, 4, 5, 16, and 12 mW, respectively). The setup includes gold parabolic mirrors to focus the incident THz beam on the sample, with the THz radiation linearly polarized parallel to the bow‐tie antenna axis. Finally, the photocurrent signal was detected as a voltage drop by using a low‐noise current‐to‐voltage preamplifier and measured via a standard lock‐in technique (THz waves were electrically modulated at a frequency of 337 Hz).

## 3 Results and Discussion

Initially, we measured the magenetotransport characteristics of our single and bilayer graphene TeraFETs via electrical measurements. Average mobilities, *μ*, in the devices exceeded 70,000 cm^2^V^−1^s^−1^ for the single‐layer and bilayer graphene TeraFETs at 1.7 K. The momentum‐relaxing scattering time of charge carriers, *τ*, can be calculated with the equation *τ = mμ/e* (where *e* is the elementary charge and *m* is the effective mass of carriers in graphene) with the values lying above 0.4 ps for both graphene systems.

We then studied the THz photoresponse in our devices at the zero magnetic field. Firstly, the devices were illuminated at frequencies between 0.246 and 0.309 THz, intentionally operating in the nonresonant (broadband) scenario (*Q* ≪ 1). As expected for the nonresonant operation regime, the photoresponse in single layer TeraFET (Figure 1c—bottom panel) and bilayer TeraFET (Supporting Information S1: Note 2) exhibits the characteristic antisymmetric shape, reversing sign at the charge neutrality point (CNP). Apart from this central feature, the signal remains monotonic, vanishing at large gate voltages, consistent with a nonresonant overdamped regime. Near the CNP, we have observed a rectified voltage response that is slightly larger on the hole side than the electron side, reflecting the superior hole carrier mobility in our device.

The scenario drastically changes when the excitation frequency is increased into the high THz range, as the resonant condition (*Q* > 1) can be clearly satisfied in our graphene TeraFETs. For example, clear signatures corresponding to the appealing resonant operation in the single‐layer graphene TeraFET at 3.5 THz appear on the THz photoresponse curve with prominent oscillations arising at larger gate voltages and being dependent on the carrier density in the system and the excitation frequency (see Figure 1c top panel). These peaks stem from plasmon resonances in the graphene channel, where the channel under the gate acts as a Fabry–Perot resonant cavity causing reflections at the boundaries and giving rise to standing plasma waves through constructive interferences of counter‐propagating modes. Their emergences mark the transition to a resonant regime, in contrast to the nonresonant behavior observed at lower frequencies. For completeness, we additionally measured the THz photoresponse in both systems, single‐layer (Figure 2a) and bilayer (Figure 2b) TeraFETs, within the range of 2.5–3.8 THz, all within the resonant regime or the weakly damped scenario (*Q* > 1). For simplicity and clarity, we only present the photoresponse evolution close to the CNP at the electron side. In this framework, excited plasmons in two‐dimensional systems are characterized by their angular frequency, $\omega_{p}$, which can be written as

(1) $$\omega_{p}=sk,$$

where *k* is the real part of the wave vector [25, 26] and *s* is the plasma‐wave velocity defined as

(2) $$s=\sqrt{\frac{e}{m*}\left|V_{\text{TG}}\right|},$$

with m* being the effective electron mass, *e* the elementary carrier charge, and $V_{\text{TG}}$ the top gate voltage. The multiple peaks correspond to the different resonant modes occurring when the wave number satisfies the following condition:

(3) $$k=\frac{\pi }{2L_{\text{TG}}}\left(2N+1\right),N=0,1,2,\text{…},$$

where, *L*_TG_ is the length of the gated channel (plasmonic Fabry–Perot cavity) taken as a first‐order approach of the effective plasmonic cavity length (possible corrections associated with the ungated access regions and boundaries may lead to small corrections) and *N* denotes the resonance mode number. Importantly, the effective mass m* in Equation (2) must be carefully defined, as its value is different in single‐layer or bilayer graphene. On one side, m* is defined in a single‐layer graphene [26, 42] as

(4) $$m^{*}=\frac{\hbar }{v_{F}}\sqrt{\frac{\pi C_{\text{TG}}\left|V_{\text{TG}}\right|}{e}},$$

with *C*_TG_ being the thin‐hBN top‐gate capacitance per unit area (calculated value for our TeraFET system is 0.00112 F.m^−2^, see Supporting Information S1: Note 3) and *v*_*F*_ the Fermi velocity of the charge carriers (1.19·10^6^ m·s^−1^, as described by Chae et al. [43]). In contrast, for the bilayer graphene, following the conventional Schrödinger‐based picture, experimental data were fitted with an effective mass of ∼0.026 *m*_0_, which agrees well with values reported in the literature [44]. As a result, the plasma frequency ($f_{p}=\frac{\omega_{p}}{2\pi }$) in monolayer graphene‐based TeraFETs takes the following form:

(5) $$f_{p}^{\text{mono}}=\frac{1}{4L_{\text{TG}}}\left(2N+1\right)\sqrt{\frac{v_{F}}{\hbar }}{\left(\frac{e^{3}\left|V_{\text{TG}}\right|}{\pi C_{\text{TG}}}\right)}^{\frac{1}{4}},$$

whereas for the bilayer graphene TeraFETs, the plasma frequency takes the following form:

(6) $$f_{p}^{\text{bi}}=\frac{1}{4L_{\text{TG}}}\left(2N+1\right)\sqrt{\frac{e}{m*}\left|V_{\text{TG}}\right|}.$$

**FIGURE 2:**
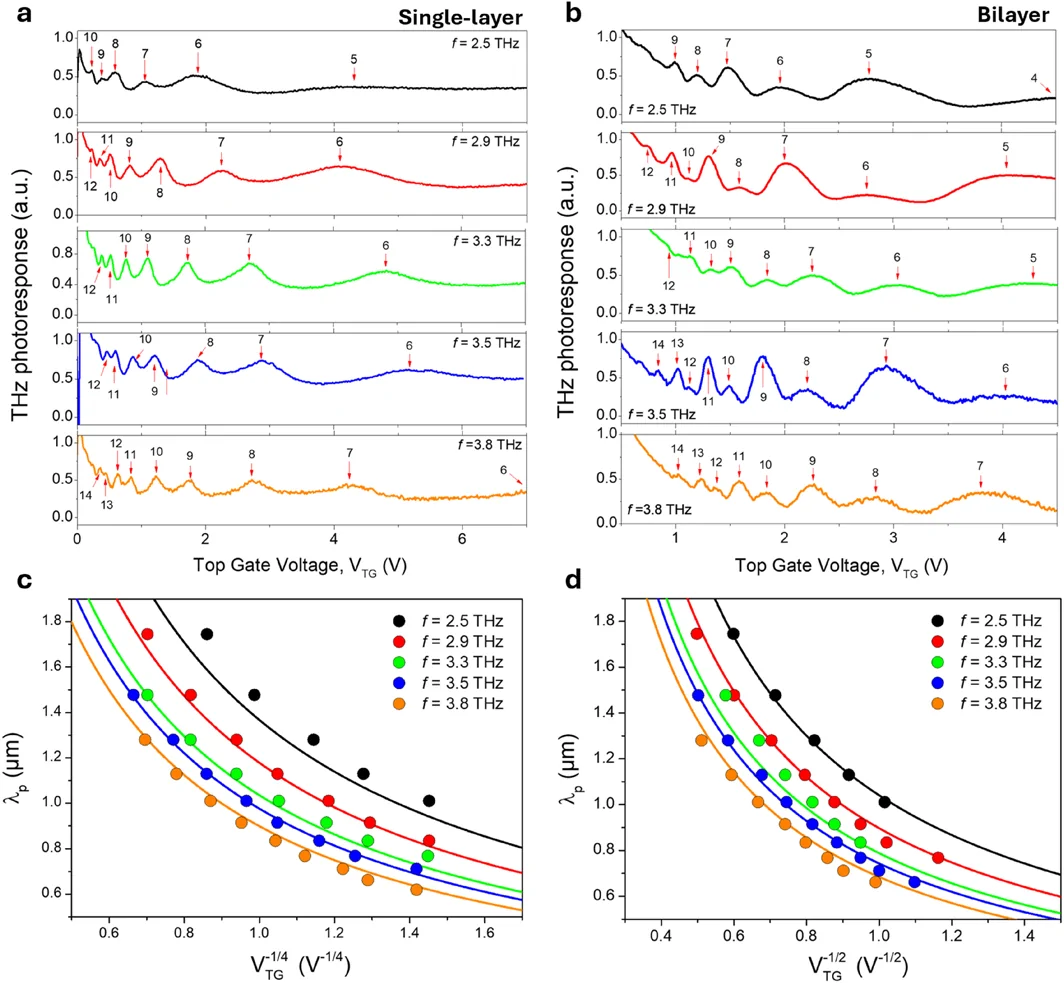
Onset of the plasma‐wave resonant regime. Photoresponse versus top‐gate voltage measured at frequencies ranging from 2.5 THz to 3.8 THz, performed at 1.7 K for monolayer (a) and at 15K for bilayer (b) graphene‐based TeraFETs, respectively. Resonant plasmon mode peaks, marked by red arrows, are labeled in Equations (3), (5), and (6). Plasmon wavelengths, *λ*_p_, are plotted as a function of |VTG|−14 for the monolayer device (c) and as a function of |VTG|−12 for the bilayer device (d) for illumination frequencies at 2.5, 2.9, 3.3, 3.5, and 3.8 THz with respective symbols. *λ*_p_ = 2π/k has been calculated based on the top‐gate voltage values where the peaks emerged in panels (b) and (c) as shown in Equations (5) and (6). The observed trends show good agreement with the Dyakonov–Shur theory adapted by monolayer graphene and bilayer graphene (solid lines).

The resonant peaks observed in Figure 2a and b correspond to modes defined by Equations (5) and (6) for the monolayer and bilayer graphene, respectively, with their order set by the plasmonic cavity length (top‐gate length) and the applied gate voltage $V_{\text{TG}}$. As the frequency increases, more plasmon modes become accessible, because higher frequencies correspond to shorter wavelengths that can fit within the same channel length [25, 26]. From the modal structure, the plasmon wavelength can be extracted using *λ*_p_ = 2π/k [25, 26]. Figure 2c and d show the extracted wavelength as a function of |VTG|−14 for the monolayer graphene and |VTG|−12 for the bilayer graphene, respectively, in good agreement with the expected behavior for both systems. Similarly, one can plot the N mode number of the observed plasmons as a function of |VTG|−14 for the monolayer graphene and |VTG|−12 for the bilayer graphene highlighting a clear linear dispersion in both systems (see Supporting Information S1: Note 4). The observed evolution exhibits unambiguous signatures of resonant operation consistent with Dyakonov–Shur plasma‐wave theory [7, 25, 26, 42], demonstrating the presence of resonant plasma modes in the gated plasmonic cavity and enabling a direct comparison between monolayer and bilayer graphene systems—materials with distinct plasmon dispersions—under identical excitation and boundary conditions. Beyond dispersion, the resonance linewidth also provides access to the plasmon lifetime, *τ*_*p*_, which can be extracted from Lorentzian fits to the observed peaks in the THz photoresponse curves. The obtained values (typically above 1 ps) slightly exceed those found in the transport times, indicating that momentum scattering alone does not set the linewidth (see Supporting Information S1: Note 5).

Building on the zero‐field measurements presented above, we have investigated the effect of a perpendicular variable magnetic field on the plasmonic resonances. For a 3.5‐THz illumination, increasing the magnetic field causes a clear shift of the resonance peaks toward lower carrier densities (i.e., lower gate voltage), as shown in Figure 3 and Supporting Information S1: Figure S6 (see Supporting Information S1: Note 6). This shift is more pronounced for higher order modes than for those near the CNP, indicating a mode‐dependent sensitivity to the magnetic field. Such behavior results from the coupling between plasma waves and the cyclotron motion of charge carriers, leading to the formation of magnetoplasmon modes. At higher magnetic fields, additional oscillations emerge near the CNP, consistent with Shubnikov–de Haas (SdH) oscillations, which become increasingly prominent and progressively obscure the magnetoplasmon peaks.

**FIGURE 3:**
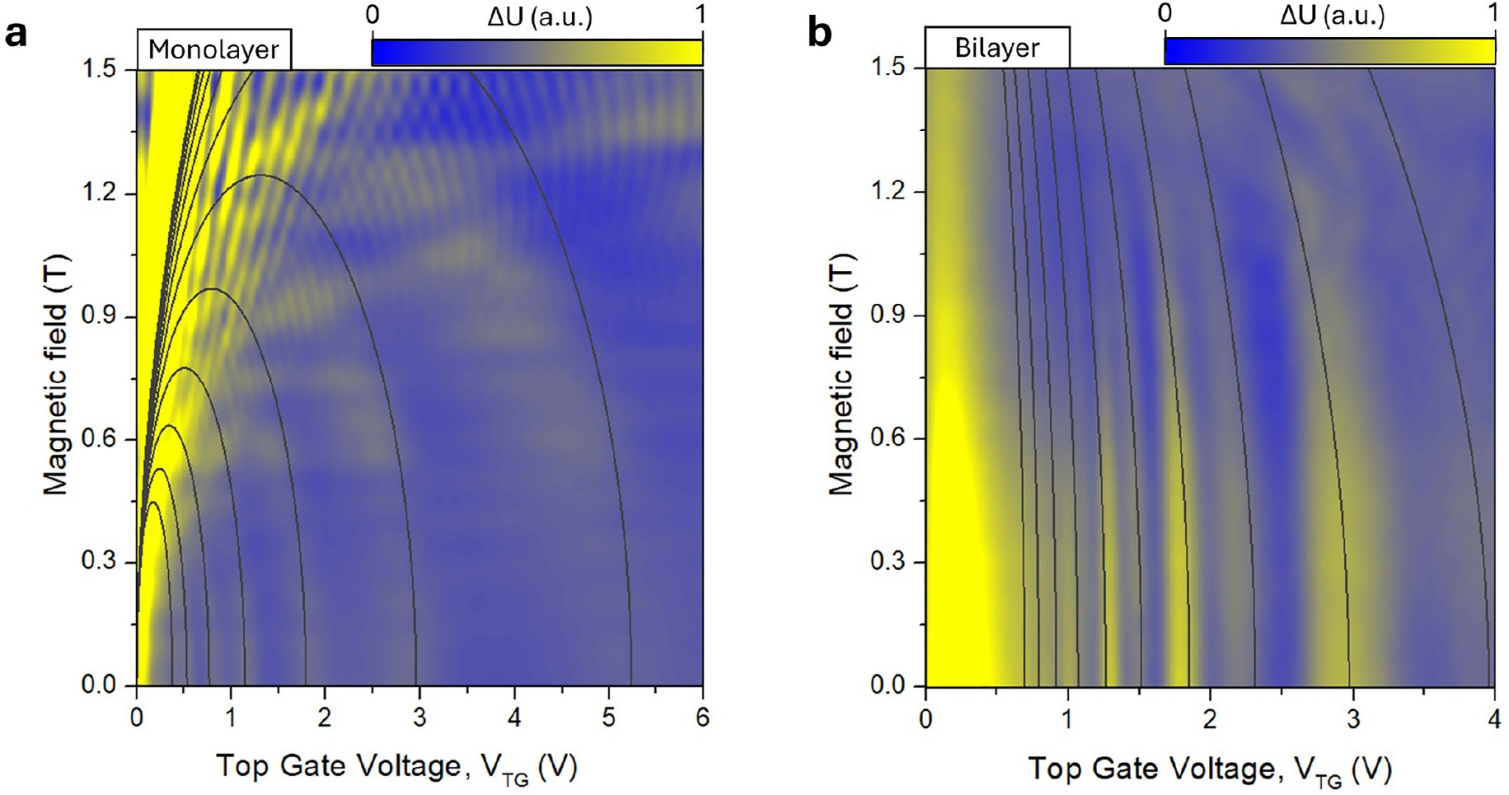
Resonant magnetoplasmons in graphene TeraFETs. Mapping of the photoresponse as a function of the magnetic field and top‐gate voltage under 3.5 THz illumination, measured at 1.7 K for monolayer graphene (a) and at 15 K for bilayer graphene (b). The yellow color indicates those values with higher intensity measured in the THz photoresponse mapping, whereas the blue color indicates the lower values. Theoretical curves for the magnetoplasmon resonance positions are shown in gray lines in both mappings, calculated using Equation (10) for the Dirac (monolayer, panel (a)) system and Equation (11) for the Schrödinger (bilayer, panel (b)) system.

In the long‐wavelength limit, the dispersion of two‐dimensional magnetoplasmons can be expressed with the following relation [45]:

(7) $$\omega_{\text{mp}}=\sqrt{\omega_{p}^{2}+\omega_{c}^{2}},$$

where $\omega_{c}$ is the cyclotron angular frequency given by eB/(m∗) and $\omega_{p}$ is the plasma‐wave frequency at the zero magnetic field, determined by Equations (5) and (6) for systems with linear or parabolic bands, respectively. As such, according to the Dyakonov–Shur theory presented above and adapted to monolayer graphene, the frequency of the Dirac magnetoplasmon resonance takes the following form:

(8) $$f_{\text{mp}}^{D}=\frac{1}{2\pi }\sqrt{\frac{e^{3/2}v_{F}}{\hbar }{\left(\frac{\left|V_{\text{TG}}\right|}{\pi C_{\text{TG}}}\right)}^{1/2}{\left(\frac{\pi }{2L_{\text{TG}}}\left(2N+1\right)\right)}^{2}+{\left(\frac{Bv_{f}}{\hbar }\right)}^{2}\frac{e^{3}}{\pi C_{\text{TG}}\left|V_{\text{TG}}\right|}}.$$

The gate‐voltage dependence of the effective mass given in Equation (4) for monolayer graphene leads to a bulk magnetoplasmon resonance frequency with two terms explicitly proportional to $V_{\text{TG}}$. In a clear contrast, the magnetoplasmon resonance frequency for the case of bilayer graphene is given by a simpler expression, derived from the Schrödinger equation with an effective mass description as follows:

(9) $$f_{\text{mp}}^{S}=\frac{1}{2\pi }\sqrt{\left(\frac{e}{m*}\left|V_{\text{TG}}\right|\right){\left(\frac{\pi }{{2L}_{\text{TG}}}\left(2N+1\right)\right)}^{2}+{\left(\frac{eB}{m*}\right)}^{2}},$$

where the bulk magnetoplasmon resonance frequency contains only a single term dependent on $V_{\text{TG}}$. In our measurements, graphene TeraFETs are exposed to THz radiation at a fixed frequency ($f_{\text{source}}$). Thus, gate voltage is “tuned” in order to satisfy to the bulk magnetoplasmon resonance condition $f_{\text{mp}}=f_{\text{source}}$. Under this condition, Equation (7) can be rearranged to express the magnetic field as a function of the top‐gate voltage $V_{\text{TG}}$. In the case of the Dirac magnetoplasmon in monolayer graphene, this relation takes the following form:

(10) $$B^{D}=\sqrt{\left[4\pi^{2}f_{\text{Source}}^{2}-\frac{e^{3/2}v_{F}}{\hbar }{\left(\frac{\left|V_{\text{TG}}\right|}{\pi C_{\text{TG}}}\right)}^{1/2}{\left(\frac{\pi }{2L_{\text{TG}}}\left(2N+1\right)\right)}^{2}\right]\frac{\pi C_{\text{TG}}\left|V_{\text{TG}}\right|}{e^{3}}}\left(\frac{\hbar }{v_{f}}\right),$$

For the so‐called Schrödinger‐type magnetoplasmon in bilayer graphene follows the form:

(11) $$B^{S}=\frac{m*}{e}\sqrt{\left[{4\pi^{2}f}_{\text{Source}}^{2}-\left(\frac{e}{m*}\left|V_{\text{TG}}\right|\right){\left(\frac{1\pi }{2L_{\text{TG}}}\left(2N+1\right)\right)}^{2}\right]},$$

Accordingly, the magnetic field where magnetoplasmon resonance arises can be represented as a function of the top‐gate voltage. Figure 3 presents a comparative analysis between Dirac‐type (monolayer) and Schrödinger‐type (bilayer) systems, highlighting the impact of the effective mass on the magnetoplasmon dispersion.

Theoretical resonance positions, obtained from Equations (10) and (11), are superimposed on the color maps of the measured THz photoresponse as a function of magnetic field and top‐gate voltage, under 3.5 THz illumination at 1.7 and 15 K, for monolayer and bilayer graphene TeraFETs, respectively. In monolayer graphene (see Figure 3a), Shubnikov–de Haas oscillations become increasingly pronounced near the CNP as the magnetic field increases, thereby hindering experimental access to the low‐density and low‐field regime and progressively obscuring the magnetoplasmon resonances. Nevertheless, the resonance that can be resolved follows the expected |VTG|−14 dependence, consistent with the Dirac model. Moreover, the analysis of the resonance indicates a nonmonotonic dependence of the resonance magnetic field (B_res_) on carrier density, as predicted by theory. This behavior indicates a crossover between two distinct dynamical regimes: at a low carrier density, the density‐dependent cyclotron response of the Dirac spectrum drives the resonance toward zero magnetic field, whereas at higher densities, the collective plasmonic response, determined by carrier density and spatial confinement, becomes dominant. This nonmonotonic behavior is a hallmark of Dirac carriers, reflecting their density‐dependent cyclotron response and distinguishing them from conventional parabolic‐band systems. The maximum in B_res_(n) at intermediate densities reveals pronounced cyclotron–plasmon hybridization, with the resonance governed by the interplay of both modes.

In contrast to the monolayer graphene, measurements in bilayer graphene revealed universal conductance fluctuations (UCF) [46], becoming prominent at temperatures below 10K, preventing the clear resolution and observation of graphene magnetoplasmon resonances. However, to suppress these phase‐coherence effects, measurements were intentionally performed at higher temperatures (15 K), which allows a clear observation of magnetoplasmons at this temperature. A comparative dataset at 1.7 K and 15 K is included in Supporting Information S1: Note 7, demonstrating that the suppression of UCF at higher temperature is required to clearly resolve the magnetoplasmon resonances. Therefore, Figure 3b shows the photoresponse mapping in bilayer graphene TeraFETs but at moderate temperatures of 15 K, modeled in the Schrödinger framework with a fixed effective mass of *m* ^∗^/*m*_0_ = 0.0026 [44]. The resonance exhibits a |VTG|−12 scaling, consistent with a Schrödinger‐type description. In this case, the resonance tends toward the cyclotron frequency at a low carrier density, as expected for systems characterized by an effective mass. For both monolayer and bilayer graphene, the agreement with theoretical predictions extends over a wide frequency range, from 1 THz to 3.8 THz, as shown in the Supporting Information S1: Note 8 although the inflection region is not resolved with equal clarity for all branches in monolayer graphene because Shubnikov–de Haas oscillations progressively obscure the low‐density finite‐field regime.

To the best of our knowledge, the only previous study closely related to the resonant Dyakonov–Shur plasma‐wave model under a perpendicular magnetic field is that of Park et al. [45], who reported magnetoplasmon resonances up to 400 GHz in gated GaAs/AlGaAs mesa structures using on‐chip THz time‐domain spectroscopy. Their work represents an important milestone in conventional III–V 2DEG systems, whereas our study advances the field by demonstrating clear signatures of cavity‐confined magnetoplasmon resonances in a fundamentally different material platform across the THz range (up to 3.8 THz). Here, we move beyond conventional III–V 2DEGs with parabolic dispersion to establish the robustness and universality of the Dyakonov–Shur magneto‐resonant regime in monolayer graphene, a unique platform with a linear, gapless Dirac band structure. In parallel, bilayer graphene offers a complementary parabolic system that, unlike GaAs‐based heterostructures, features a gate‐tunable bandgap (via displacement field), ambipolar operation with the gate bias, and low‐disorder hBN encapsulation enabling several long‐lived cavity plasmons. These attributes provide an alternative route to resonant plasmonics not accessible in conventional semiconductors and allow a direct comparison with the gapless, linear monolayer case. The observation of magnetoplasmon resonances in both mono‐ and bilayer graphene highlights not only the adaptability of the Dyakonov–Shur mechanism to emerging 2D materials, but also its relevance in regimes far from those traditionally explored in III–V heterostructures. Overall, we show a good agreement between experiment and theory in both monolayer and bilayer graphene systems. As shown in Figures 2 and 3 in the main text and also additional data presented in the Supporting Information S1: Note 8, minor deviations can be observed at *B* = 0 in both materials, despite the overall good agreement provided by the parabolic‐band model for the bilayer and the Dirac model for the monolayer. These slightly low‐field mismatches, particularly for the lowest order modes at larger $\left|V_{\text{TG}}\right|$ where the resonances are broader and the exact peak position is more difficult to determine, can reasonably be ascribed to uncertainties in electron density calibration [47], global parameters such as the Fermi velocity [48, 49] or oxide capacitance [50], as well as possible fringing effects [51]. Beyond this baseline discrepancy, the monolayer exhibits an additional deviation at the finite magnetic field. Although the initial field dependence follows perfectly theoretical prediction, the experimental curves consistently lie below the calculated ones near the inflection point, corresponding to the maximum of the resonance magnetic field. The origin of this additional shift remains uncertain. Nonlocal electrodynamics could, in principle, contribute to the effect [52, 53, 54], because incorporating nonlocal terms in the dispersion lowers the resonance maximum in qualitative agreement with experiment. However, this interpretation should be regarded as tentative, because nonlocality would also alter the dispersion at low fields, where theory and experiment otherwise agree well in some of the spectra. However, this interpretation should be regarded as tentative, because nonlocality would also alter the dispersion at low fields, where theory and experiment otherwise agree well in some of the spectra. In addition, electron–electron interactions may play a significant role. These interactions are known to renormalize the Fermi velocity, particularly at low carrier concentrations [55, 56] leading to an enhancement of *v*_*F*_ and, consequently, to a downward shift of the resonant magnetic field maximum. Finally, edge magnetoplasmons and their possible coupling to bulk modes [34] could also contribute to the observed resonance shift. Such combined effects involving geometric factors, nonlocal corrections, and many‐body interactions, need further investigation to fully elucidate the origin of the deviations observed in the magnetoplasmon dispersion of graphene.

## 4 Conclusion

In summary, we have investigated resonant Dyakonov–Shur magnetoplasmons in high‐mobility mono‐ and bilayer graphene TeraFETs subjected to THz radiation. In absence of a magnetic field, both systems present univocal fingerprints of plasmon‐assisted resonant THz photodetection, providing a direct comparison between two unique systems with distinct plasma‐wave dispersions in a wide frequency operation range (2.5–3.8 THz). For a finite magnetic field, perpendicularly applied to the systems, the observed resonant magnetoplasmon modes are due to the coupling of standing plasma‐waves under the gate with the cyclotron motion of carriers. In bilayer graphene, the magnetoplasmon dispersion is well captured by a Schrödinger‐type model with a constant effective mass, whereas in monolayer graphene, it follows the Dirac picture with a density‐dependent cyclotron response. This leads to a nonmonotonic evolution of the Dirac magnetoplasmon dispersion with carrier density, including an inflection point where the coupling between the plasmon and the cyclotron resonance is strongest. Overall, the theoretical framework reproduces the experimental dispersion well across a broad range of parameters such as gate voltage, magnetic field, and frequency. These findings establish graphene TeraFETs as a robust platform for exploring magnetoplasmons and for advancing future reconfigurable terahertz plasmonic sensing technologies.

## Author Contributions

**Juan A. Delgado‐Notario:** conceptualization, investigation, funding acquisition, writing – original draft, methodology, validation, visualization, writing – review and editing, resources, supervision. **Cedric Bray** **:** investigation, writing – original draft, methodology, validation, visualization, writing – review and editing, formal analysis, data curation. **Elsa Pérez‐Martín** **:** validation, visualization, writing – review and editing. **Benjamin Benhamou‐Bui** **:** validation, visualization, writing – review and editing. **Namrata Saha:** validation, investigation, writing – review and editing. **Sahil Parvez** **:** validation, investigation, writing – review and editing. **Christophe Consejo**: validation, visualization, writing – review and editing. **Guillaume Sigu:** validation, visualization, writing – review and editing. **Salah Benlemqwanssa:** validation, visualization, writing – review and editing. **Laurent Bonnet:** validation, visualization, writing – review and editing. **Takashi Taniguchi:** funding acquisition, writing – review and editing, resources. **Kenji Watanabe:** funding acquisition, writing – review and editing, resources. **José M. Caridad** **:** validation, investigation, writing – review and editing, funding acquisition, resources. **Sergey S. Krishtopenko:** validation, visualization, writing – review and editing. **Yahya M. Meziani:** validation, writing – review and editing, funding acquisition. **Benoit Jouault:** investigation, methodology, validation, software, writing – review and editing, formal analysis. **Jérémie Torres:** investigation, validation, visualization, writing – review and editing. **Sandra Ruffenach:** investigation, writing – original draft, methodology, validation, visualization, writing – review and editing, formal analysis, supervision, data curation. **Frédéric Teppe** **:** conceptualization, funding acquisition, writing – original draft, methodology, validation, visualization, writing – review and editing, formal analysis, project administration, supervision, resources.

## Supporting information


Supporting Information S1

## Data Availability

The data that support the findings of this study are available from the corresponding author upon reasonable request.

## References

[1] D. N. Basov , M. M. Fogler , and F. J. García De Abajo , “Polaritons in van der Waals Materials,” Science 354, no. 6309 (2016): aag1992, 10.1126/science.aag1992. 27738142

[2] C. In , U. J. Kim , and H. Choi , “Two‐Dimensional Dirac Plasmon‐Polaritons in Graphene, 3D Topological Insulator and Hybrid Systems,” Light: Science & Applications 11, no. 1 (2022): 313, 10.1038/s41377-022-01012-2. PMC9613982 36302746

[3] Y. Li , K. Tantiwanichapan , A. K. Swan , and R. Paiella , “Graphene Plasmonic Devices for Terahertz Optoelectronics,” Nanophotonics 9 (2020): 7–1920, 10.1515/nanoph-2020-0211.

[4] G. X. Ni , A. S. McLeod , Z. Sun , et al., “Fundamental Limits to Graphene Plasmonics,” Nature 557, no. 7706 (2018): 530–533, 10.1038/s41586-018-0136-9. 29795255

[5] S. Huang , C. Song , G. Zhang , and H. Yan , “Graphene Plasmonics: Physics and Potential Applications,” Nanophotonics 6 (2017): 6–1204, 10.1515/nanoph-2016-0126.

[6] T. Low and P. Avouris , “Graphene Plasmonics for Terahertz to Mid‐Infrared Applications,” ACS Nano 8 (2014): 2–1101, 10.1021/nn406627u. 24484181

[7] M. Dyakonov and M. Shur , “Detection, Mixing, and Frequency Multiplication of Terahertz Radiation by Two‐Dimensional Electronic Fluid,” IEEE Trans. Electron Devices 43 (1996): 3–387, 10.1109/16.485650.

[8] M. Dyakonov and M. Shur , “Shallow Water Analogy for a Ballistic Field Effect Transistor: New Mechanism of Plasma Wave Generation by Dc Current,” Physical Review Letters 71, no. 15 (1993): 2465–2468, 10.1103/PhysRevLett.71.2465. 10054687

[9] J. Calvo‐Gallego , J. A. Delgado‐Notario , O. V. Minin , et al., “Enhancing Resolution of Terahertz Imaging Systems Below the Diffraction Limit,” Optics & Laser Technology 164 (2023): 109540, 10.1016/j.optlastec.2023.109540.

[10] W. Knap , V. Kachorovskii , Y. Deng , et al., “Nonresonant Detection of Terahertz Radiation in Field Effect Transistors,” Journal of Applied Physics 91, no. 11 (2002): 9346–9353, 10.1063/1.1468257.

[11] E. Javadi , J. A. Delgado‐Notario , N. Masoumi , M. Shahabadi , J. E. Velázquez‐Pérez , and Y. M. Meziani , “Continuous Wave Terahertz Sensing Using GaN HEMTs,” Physica Status Solidi (a) 215, no. 11 (2018): 1700607, 10.1002/pssa.201700607.

[12] A. M. Kadykov , F. Teppe , C. Consejo , et al., “Terahertz Detection of Magnetic Field‐Driven Topological Phase Transition in HgTe‐Based Transistors,” Applied Physics Letters 107, no. 15 (2015): 56, 10.1063/1.4932943.

[13] J. A. Delgado‐Notario , W. Knap , V. Clericò , et al., “Enhanced Terahertz Detection of Multigate Graphene Nanostructures,” Nanophotonics 11, no. 3 (2022): 519–529, 10.1515/nanoph-2021-0573. PMC11501572 39633800

[14] L. Vicarelli , M. S. Vitiello , D. Coquillat , et al., “Graphene Field‐Effect Transistors as Room‐Temperature Terahertz Detectors,” Nature Materials 11, no. 10 (2012): 865–871, 10.1038/nmat3417. 22961203

[15] J. A. Delgado‐Notario , S. R. Power , W. Knap , et al., “Unveiling the Miniband Structure of Graphene Moiré Superlattices via Gate‐Dependent Terahertz Photocurrent Spectroscopy,” ACS Nano 19 (2025): 30–27350, 10.1021/acsnano.5c05306. PMC12333420 40689458

[16] L. Viti , J. Hu , D. Coquillat , et al., “Black Phosphorus Terahertz Photodetectors,” Advanced 27, no. 37 (2015): 5567–5572, 10.1002/adma.201502052. 26270791

[17] E. I. Titova , M. A. Kashchenko , A. V. Miakonkikh , et al., “Non‐Saturated Performance Scaling of Graphene Bilayer Sub‐Terahertz Detectors at Large Induced Bandgap,” Advanced Optical Materials 13, no. 16 (2025): 2500167, 10.1002/adom.202500167.

[18] W. Knap , Y. Deng , S. Rumyantsev , and M. S. Shur , “Resonant Detection of Subterahertz and Terahertz Radiation by Plasma Waves in Submicron Field‐Effect Transistors,” Applied Physics Letters 81, no. 24 (2002): 4637–4639, 10.1063/1.1525851.

[19] X. G. Peralta , S. J. Allen , M. C. Wanke , et al., “Terahertz Photoconductivity and Plasmon Modes in Double‐Quantum‐Well Field‐Effect Transistors,” Applied Physics Letters 81, no. 9 (2002): 1627–1629, 10.1063/1.1497433.

[20] F. Teppe , W. Knap , D. Veksler , et al., “Room‐Temperature Plasma Waves Resonant Detection of Sub‐Terahertz Radiation by Nanometer Field‐Effect Transistor,” Applied Physics Letters 87, no. 5 (2005): 052107, 10.1063/1.2005394.

[21] F. Teppe , D. Veksler , V. Y. Kachorovski , et al., “Plasma Wave Resonant Detection of Femtosecond Pulsed Terahertz Radiation by a Nanometer Field‐Effect Transistor,” Applied Physics Letters 87, no. 2 (2005): 022102, 10.1063/1.1952578.

[22] A. El Fatimy , F. Teppe , N. Dyakonova , et al., “Resonant and Voltage‐Tunable Terahertz Detection in InGaAs∕ InP Nanometer Transistors,” Applied Physics Letters 89, no. 13 (2006): 131926, 10.1063/1.2358816.

[23] S. Boubanga‐Tombet , F. Teppe , D. Coquillat , et al., “Current Driven Resonant Plasma Wave Detection of Terahertz Radiation: Toward the Dyakonov–Shur Instability,” Applied Physics Letters 92, no. 21 (2008): 212101, 10.1063/1.2936077.

[24] G. C. Dyer , N. Q. Vinh , S. J. Allen , et al., “A Terahertz Plasmon Cavity Detector,” Applied Physics Letters 97, no. 19 (2010): 193507, 10.1063/1.3513339.

[25] D. A. Bandurin , D. Svintsov , I. Gayduchenko , et al., “Resonant Terahertz Detection Using Graphene Plasmons,” Nature Communications 9, no. 1 (2018): 5392, 10.1038/s41467-018-07848-w. PMC6300605 30568184

[26] J. M. Caridad , Ó. Castelló , S. M. López Baptista , et al., “Room‐Temperature Plasmon‐Assisted Resonant THz Detection in Single‐Layer Graphene Transistors,” Nano Letters 24 (2024): 3–942, 10.1021/acs.nanolett.3c04300. PMC10811671 38165655

[27] L. F. Man , W. Xu , Y. M. Xiao , et al., “Terahertz Magneto‐Optical Properties of Graphene Hydrodynamic Electron Liquid,” Physical Review B: Condensed Matter 104, no. 12 (2021): 125420, 10.1103/PhysRevB.104.125420.

[28] K. Kapralov and D. Svintsov , “Ballistic‐to‐Hydrodynamic Transition and Collective Modes for Two‐Dimensional Electron Systems in Magnetic Field,” Physical Review B: Condensed Matter 106, no. 11 (2022): 115415, 10.1103/PhysRevB.106.115415.

[29] Q. Shen , J. Yan , Y. You , S. Li , and L. Shen , “Terahertz Large‐Area Unidirectional Surface Magnetoplasmon and Its Applications,” Scientific Reports 13, no. 1 (2023): 21945, 10.1038/S41598-023-49348-Y. PMC10713579 38082053

[30] S. Chandra , J. Cozart , A. Biswas , S. Lee , and D. Chanda , “Magnetoplasmons for Ultrasensitive Label‐Free Biosensing,” ACS Photonics 8, no. 5 (2021): 1316–1323, 10.1021/acsphotonics.0c01646.

[31] J. M. Poumirol , W. Yu , X. Chen , et al., “Magnetoplasmons in Quasineutral Epitaxial Graphene Nanoribbons,” Physical Review Letters 110, no. 24 (2013): 246803, 10.1103/PhysRevLett.110.246803. 25165953

[32] I. Crassee , M. Orlita , M. Potemski , et al., “Intrinsic Terahertz Plasmons and Magnetoplasmons in Large Scale Monolayer Graphene,” Nano Letters 12 (2012): 5–2474, 10.1021/nl300572y. 22519967

[33] H. Bartolomei , R. Bisognin , H. Kamata , et al., “Observation of Edge Magnetoplasmon Squeezing in a Quantum Hall Conductor,” Physical Review Letters 130, no. 10 (2023): 106201, 10.1103/PhysRevLett.130.106201. 36962050

[34] H. Yan , Z. Li , X. Li , W. Zhu , P. Avouris , and F. Xia , “Infrared Spectroscopy of Tunable Dirac Terahertz Magneto‐Plasmons in Graphene,” Nano Letters 12 (2012): 7–3771, 10.1021/NL3016335. 22690695

[35] D. A. Bandurin , E. Mönch , K. Kapralov , et al., “Cyclotron Resonance Overtones and Near‐Field Magnetoabsorption via Terahertz Bernstein Modes in Graphene,” Nature Physics 18, no. 4 (2022): 462–467, 10.1038/s41567-021-01494-8.

[36] M. H. Eriksen , J. R. Deop‐Ruano , J. D. Cox , and A. Manjavacas , “A. Chiral Light–Matter Interactions With Thermal Magnetoplasmons in Graphene Nanodisks,” Nano Letters 25 (2025): 1, 10.1021/acs.nanolett.4c05056. 39718382

[37] X. G. Peralta , S. J. Allen , N. E. Harff , et al., “Terahertz Photoconductivity and Magnetoplasmons in Double Quantum Wells,” American Physical Society—Annual March Meeting, March 12 – 16 (2001), https://ui.adsabs.harvard.edu/abs/2001APS..MARN30006P.

[38] E. Frigerio , G. Rebora , M. Ruelle , et al., “Gate Tunable Edge Magnetoplasmon Resonators,” Communications Physics 7, no. 1 (2024): 314, 10.1038/S42005-024-01803-6.

[39] N. Hiyama , M. Hashisaka , and T. Fujisawa , “An Edge‐Magnetoplasmon Mach‐Zehnder Interferometer,” Applied Physics Letters 107, no. 14 (2015): 143101, 10.1063/1.4932111.

[40] W. Zhao , S. Wang , S. Chen , et al., “Observation of Hydrodynamic Plasmons and Energy Waves in Graphene,” Nature 614, no. 7949 (2023): 688–693, 10.1038/s41586-022-05619-8. 36813893

[41] B. Winstanley , H. Schomerus , and A. Principi , “Corbino Field‐Effect Transistors in a Magnetic Field: Highly Tunable Photodetectors,” Physical Review B: Condensed Matter 104, no. 16 (2021): 165406, 10.1103/PhysRevB.104.165406.

[42] A. Tomadin and M. Polini , “Theory of the Plasma‐Wave Photoresponse of a Gated Graphene Sheet,” Physical Review B: Condensed Matter 88, no. 20 (2013): 205426, 10.1103/PhysRevB.88.205426.

[43] J. Chae , S. Jung , A. F. Young , et al., “Renormalization of the Graphene Dispersion Velocity Determined From Scanning Tunneling Spectroscopy,” Physical Review Letters 109, no. 11 (2012): 116802, 10.1103/PhysRevLett.109.116802. 23005662

[44] K. Zou , X. Hong , and J. Zhu , “Effective Mass of Electrons and Holes in Bilayer Graphene: Electron‐Hole Asymmetry and Electron‐Electron Interaction,” Physical Review B: Condensed Matter 84, no. 8 (2011): 085408, 10.1103/PhysRevB.84.085408.

[45] J. E. Cunningham , E. H. Linfield , C. D. Wood , et al., “Terahertz Magnetoplasmon Resonances in Coupled Cavities Formed in a Gated Two‐Dimensional Electron Gas,” Optics Express 29 (2021): 9, 10.1364/OE.414178. 33985041

[46] M. Y. Kharitonov and K. B. Efetov , “Mesoscopic Conductance Fluctuations in Graphene Samples,” Physical Review B: Condensed Matter 78, no. 3 (2008): 033404, 10.1103/PhysRevB.78.033404.

[47] F. Xia , T. Mueller , Y. M. Lin , A. Valdes‐Garcia , and P. Avouris , “Ultrafast Graphene Photodetector,” Nature Nanotechnology 4, no. 12 (2009): 839–843, 10.1038/nnano.2009.292. 19893532

[48] D. C. Elias , R. V. Gorbachev , A. S. Mayorov , et al., “Dirac Cones Reshaped by Interaction Effects in Suspended Graphene,” Nature Physics 7, no. 9 (2011): 701–704, 10.1038/nphys2049.

[49] P. R. Whelan , Q. Shen , B. Zhou , et al., “Fermi Velocity Renormalization in Graphene Probed by Terahertz Time‐Domain Spectroscopy,” 2D Materials 7 (2020): 3, 10.1088/2053-1583/ab81b0.

[50] Z. Fei , A. S. Rodin , G. O. Andreev , et al., “Gate‐Tuning of Graphene Plasmons Revealed by Infrared Nano‐Imaging,” Nature 486, no. 7405 (2012): 82–85, 10.1038/nature11253. 22722866

[51] T. Nishimura , N. Magome , I. Khmyrova , T. Suemitsu , W. Knap , and T. Otsuji , “Analysis of Fringing Effect on Resonant Plasma Frequency in Plasma Wave Devices,” Japanese Journal of Applied Physics 48, no. 4S (2009): 04C096, 10.1143/JJAP.48.04C096.

[52] J. Pack , B. J. Russell , Y. Kapoor , et al., “Broken Symmetries and Kohn’s Theorem in Graphene Cyclotron Resonance,” Physical Review X 10, no. 4 (2020): 041006, 10.1103/PhysRevX.10.041006.

[53] E. I. Kiselev and J. Schmalian , “Nonlocal Hydrodynamic Transport and Collective Excitations in Dirac Fluids,” Physical Review B: Condensed Matter 102, no. 24 (2020): 245434, 10.1103/PhysRevB.102.245434.

[54] P. Rodriguez‐Lopez , J. S. Wang , and M. Antezza , “Electric Conductivity in Graphene: Kubo Model Versus a Nonlocal Quantum Field Theory Model,” Physical Review B: Condensed Matter 111, no. 11 (2025): 115428, 10.1103/PhysRevB.111.115428.

[55] G. L. Yu , R. Jalil , B. Belle , et al., “Interaction Phenomena in Graphene Seen Through Quantum Capacitance,” Proceedings of the National Academy of Sciences of the United States of America 110 (2013): 9–3286, 10.1073/PNAS.1300599110. PMC3587260 23401538

[56] C. Faugeras , S. Berciaud , P. Leszczynski , et al., “Landau Level Spectroscopy of Electron‐Electron Interactions in Graphene,” Physical Review Letters 114, no. 12 (2015): 126804, 10.1103/PhysRevLett.114.126804. 25860767

