# Supplementary material for: Resonant Dyakonov–Shur Magnetoplasmons in Graphene Terahertz Photodetectors
Source: Nanophotonics. 2026 Apr 21;15(8):e70094. doi: 10.1002/nap2.70094 (PMC13642158; doi:10.1002/nap2.70094)
Supplement: Supplementary file 1 [file NAP2-15-e70094-s001.docx]

Supporting Information

Resonant Dyakonov–Shur Magnetoplasmons in Graphene Terahertz Photodetectors

Juan A. Delgado-Notario^1,2┴^*, Cedric Bray^3┴^, Elsa Perez-Martin^3^, Benjamin Benhamou-Bui^3^, Namrata Saha^1,2^, Sahil Parvez^1,2^, Christophe.Consejo^3^, Guillaume Sigu^3^, Salah Benlemqwanssa^3^, Laurent Bonnet^3^, Takashi Taniguchi^4^, Kenji Watanabe^5^, José M. Caridad^1,2^, Sergey S. Krishtopenko^3^, Yahya M. Meziani^1^, Benoit Jouault^3^, Jérémie Torres^3^, Sandra Ruffenach^3^ and Frédéric Teppe^3^*

^1^ Departamento de Física Aplicada, Universidad de Salamanca, 37008 Salamanca, Spain

^2^ Unidad de Excelencia en Luz y Materia Estructurada (LUMES), Universidad de Salamanca, Salamanca 37008, Spain

^3^ L2C (UMR 5221), Université de Montpellier, CNRS, Montpellier, France

^4^ Research Center for Materials Nanoarchitectonics, National Institute for Materials Science, 1-1 Namiki, Tsukuba 305-0044, Japan

^5^ Research Center for Electronic and Optical Materials, National Institute for Materials Science, 1-1 Namiki, Tsukuba 305-0044, Japan

* juanandn@usal.es, frederic.teppe@umontpellier.fr

^┴^ These authors contributed equally

**Supporting information Note 1: Device fabrication**

All devices were fabricated on highly doped Si substrates (serving as a global back gate) coated with 300 nm thermally grown SiO₂. The fabrication flow for mono- and bilayer graphene TeraFETs was identical. First, graphite and hBN crystals were exfoliated using adhesive tape and assembled into hBN/graphene/hBN heterostructures (either mono- or bilayer graphene) by a van der Waals dry-stacking technique^[1,2]^. For this step, we intentionally selected elongated and relatively narrow (4 to 6 µm) graphene flakes of nearly constant width (**Figure S1a**) together with wider hBN crystals (**Figure S1b**). This selection facilitates defining the top-gate metallic contact and the channel width without chemical patterning of the graphene heterostructure (**Figure S1c**) and thereby preserving the high intrinsic quality of the selected graphene flakes^[3]^.

The resulting stacks (based on monolayer or bilayer graphene) were then patterned by electron-beam lithography (EBL) to define and open the contact windows to the graphene, electrodes and antenna lobes, using PMMA (6% in chlorobenzene) as the resist. The heterostructures were subsequently dry-etched in an ICP–RIE (Oxford PlasmaPro Cobra 100) with SF₆ (40 sccm, 6 mTorr) at 75 W and 10 °C, followed by e-beam evaporation of Cr/Au (3.5/55 nm) to create the metal contacts/electrodes. Finally, a second EBL step was used to define the top gate, followed by e-beam evaporation of Cr/Au (5/45 nm).

**Figure S1. Graphene based heterostructures.** Optical image of the typical exfoliated flakes of **a,** Graphene, **b,** hBN and **c,** the fabricated half-heterostructure where the dashed red lines highlight the graphene flake area with constnt width selected for the device fabrication. The hBN flake (highlighted with dashed black lines) was intentionally selected to fully cover the graphene flake.

**Supporting information Note 2: Broadband operation in Bilayer graphene TeraFETs**

**Figure S2. Bilayer Graphene-based TeraFET photodetector.** **a**, Optical microscope image of the bilayer graphene TeraFET, featuring a bow-tie antenna integrated. The right panel provides a zoomed-in view of the device, with Source, Top gate, and Drain contacts labeled. **b**, THz photoresponse as a function of the top gate voltage, V_TG_, measured for the bilayer graphene TeraFET operating in the non-resonant or broadband regime at 0.246 THz for a temperature of 1.7 K and zero magnetic field.

**Supporting information Note 3: Determination of the top-gate capacitance per unit area**

The top-gate capacitance per unit area was calculated by the formula:

$C_{TG}=\frac{\varepsilon_{hBN}\varepsilon_{0}}{t_{hBN}}$	(S1)

Where $\varepsilon_{hBN}$ is the relative permittivity of hBN, $\varepsilon_{0}$is the vacuum permittivity and $t_{hBN}$ the hBN thickness is approximately close to 28 nm in our monolayer and bilayer graphene-based devices. Using this value, a total gate capacitance of 0.00112 F.m^-2^ can be found. In addition, the hBN gate capacitance per unit area can be determinate by the Shubnikov–de Haas oscillations^[4]^. The effective capacitance between the top gate and the graphene-based channel is given by:

$C_{eff}=\frac{C_{q}}{C_{q}+C_{TG}+C_{BG}}\times C_{TG}$	(S2)

where$C_{q}$ is the quantum capacitance and $C_{TG}$ and $C_{BG}$ are the capacitance of top gate and back gate respectively. To a first approximation, we can simplify equation S2 as $C_{eff}$ ~ $C_{TG}$. This is confirmed by the periodicity of the Shubnikov–de Haas oscillations. As such, we can determinate the value of the hBN gate capacitance per unit area by the equation:

$\Delta N=\frac{g_{v}eB}{\pi\hbar}=\frac{C_{TG}\Delta V_{TG}}{e}$	(S3)

with $\Delta N$ the maximum number of electrons per unit area that each Landau level can accommodate, $g_{v}$ the valley degeneracy in graphene and $\Delta V_{TG}$ the increment of gate voltage necessary to create $\Delta N$ at the value of magnetic field, *B,* and equals the spacing between adjacent oscillation peaks. For example, in our case we obtained a value of 0.00101 F.m^-2^ for the monolayer graphene. The slight discrepancy of a few percent below the theoretical value $C_{TG}$ of is easily explained if one takes into account the full equation S2, including the prefactor $\frac{C_{q}}{C_{q}+C_{TG}+C_{BG}}$.

**Supporting information Note 4: Plasma wave resonant regime**

**Figure S3. Plasmon dispersion.** Resonant mode number of plasmons as a function of $\left| V_{TG} \right|^{\frac{-1}{4}}$ for monolayer device, **a**, and as function of $\left| V_{TG} \right|^{\frac{-1}{2}}$ for bilayer device, **b**, for illumination frequencies at 2.5, 2.9, 3.3, 3.5 and 3.8 THz (dot symbols). The observed trends show good agreement with the Dyakonov–Shur theory adapted to monolayer graphene and bilayer graphene (solid lines).

**Supporting information Note 5: Plasmon lifetime in monolayer and bilayer graphene**

The plasmon lifetime, $\tau_{p}$, can be related to the full width at half maximum (FWHM) of each resonant peak, $\delta$, observed in the photoresponse spectra via^[5,6]^:

$\frac{{V_{G}}^{-1/4}}{\delta}=\omega\tau_{p}$, for monolayer graphene	(S4)

$\frac{{V_{G}}^{-1/2}}{\delta}=\omega\tau_{p}$, for bilayer graphene	(S5)

where 𝜔 is the angular resonance frequency. To extract $\tau_{p}$, we perform simultaneous (global) Lorentzian fits of the set of resonant peaks observed in the THz photoresponse measurements for both monolayer and bilayer TeraFETs (**Figures S4 and S5**), according to the scaling across modes presented in **Equations S4** and **S5** respectively. The resulting lifetimes range from 0.4 to 1.7 ps in both systems. Consistently with previous works^[5,6]^, these values are slightly longer than the transport time values extracted from mobility, showing that momentum relaxation alone cannot account for the observed broadening. This points to the presence of additional dissipation mechanisms, such as plasmon leakage into contacts^[7]^, oblique plasma modes^[8]^, and electronic viscosity^[9]^. Nevertheless, electronic viscosity is expected to lower the plasmon lifetime near the charge neutrality point, which does not match the behaviour observed in **Figures S4 and S5**, making it unlikely to play a major role here (we consistently observe a light enhancement of plasmon lifetimes as the charge-neutrality point is approached, which coincides with the regime where higher-order resonances are resolved.). Although hydrodynamic effects can be ruled out as the origin of the observed broadening, temperature-dependent studies would still be highly valuable to investigate in more detail the influence of electron–electron interactions. In monolayer graphene, however, our measurements showed no appreciable variation of the plasmon lifetime between 1.7 K and 15 K (see **Figure S4**). Moreover, modifying the channel geometry, as shown for example in Ref. [8] and Ref. [10], offers a promising route to suppress oblique plasma modes. Such suppression has already been demonstrated to reduce the resonance linewidth in multichannel HEMTs, and applying a similar strategy to graphene could help separate extrinsic effects from the intrinsic plasmon damping mechanisms. Finally, we have observed how the uncertainty increases for higher-order modes (i.e., at lower top-gate voltages), reflecting their reduced signal-to-noise after removing the non-resonant contribution.

**Figure S4. Plasmon lifetime in monolayer graphene**. **a**, Experimental THz photoresponse for the monolayer graphene TeraFET as a function of ${V_{TG}}^{-1/4}$ (black line) upon removing the smooth non-resonant background at 1.7K for an excitation frequency of 3.5 THz. Solid blue corresponds to the global Lorentzian fit of the experimental data. **b**, Plasmon lifetime, $\tau_{p}=\frac{{V_{G}}^{-1/4}}{\delta\omega}$, as a function of the top gate voltage calculated for each mode by using the full widths at half maximum values, δ, extracted from the multi-Lorentzian fit displayed in panel **a**. Dashed grey line has been included to guide the eye. Panels **c** and **d** show the same measurements as **a** and **b**, respectively, but at 15K.

**Figure S5. Plasmon lifetime in bilayer graphene**. **a**, Experimental THz photoresponse in the bilayer graphene as a function of ${V_{TG}}^{-1/2}$ (black line) upon removing the smooth non-resonant background at 15K for an excitation frequency of 3.5 THz. Solid blue corresponds to the global Lorentzian fit of the experimental data. **b**, Plasmon lifetime, $\tau_{p}=\frac{{V_{G}}^{-1/2}}{\delta\omega}$, as a function of the top gate voltage calculated for each mode by using the full widths at half maximum values, δ, extracted from the multi-Lorentzian fit displayed in panel **a**. Dashed grey line has been included to guide the eye.

**Supporting information Note 6: Magnetoplasmons in monolayer graphene**

**Figure S6. Magnetoplasmons in monolayer graphene.** Gate-voltage dependence of the magnetoplasmon photoresponse measured at 1.7 K with 3.5 THz excitation under a perpendicular magnetic field up to 1.3 T. Curves have been vertical shifted for clarity. Close to the CNP, clear Shubnikov–de Haas oscillations arise at higher magnetic fields. Magnetoplasmon-related peaks (indicated by red arrows) shift progressively toward lower carrier densities (i.e., lower V_TG_) as the magnetic field increases. This shift is more pronounced for lower-order modes.

**Supporting information Note 7: Universal conductance fluctuations in bilayer graphene**

**Figure S7. Temperature dependence photoresponse in bilayer graphene**. Photoresponse as a function of the top gate voltage in the bilayer TeraFET for an illumination of 3.5 THz at 0.75T for a temperature of 1.7K (black curve) and 15K (red curve). The signal of photoresponse for the measurement at 15K has been multiplied by a factor x2 for clarity. The fine features observed at 1.7K arises from mesoscopic conductance fluctuations rather than noise. These fluctuations are thermally suppressed at 15K, making the plasmonic resonances visible.

**Supporting information Note 8: Additional frequency dependence data**

Observation of magnetoplasmons have been done on monolayer and bilayer graphene devices for frequencies between 2.5 THz and 3.8 THz. For the case of monolayer graphene TeraFETs, the temperature during the experimental measurement was set to 1.7K. In contrast, for the case of bilayer graphene TeraFETs, the operation temperature during the measurements was set to 15K to suppress UFCs.

**Figure S8. Frequency dependence of magnetoplasmon.** Mapping of the THz photoresponse as a function of magnetic field and the applied top-gate voltage under excitation frequencies of 2.5, 2.9, 3.3 and 3.8 THz measured at 1.7 K for monolayer graphene, **a**, and at 15K for bilayer graphene, **b**. Theoretical curves for the magnetoplasmon resonance positions are shown in gray dashed lines, calculated using Eq. (10) for the Dirac (monolayer) system and Eq. (11) for the Schrödinger (bilayer) system, as detailed in the main manuscript. A good qualitative agreement is observed between the experimental data and the respective theoretical predictions for both systems.

**References for Supporting Information**

1.	P. J. Zomer, M. H. D. Guimarães, J. C. Brant, et al., “Fast Pick up Technique for High Quality Heterostructures of Bilayer Graphene and Hexagonal Boron Nitride”, *Appl. Phys. Lett*., 105, 013101 (2014). <https://doi.org/10.1063/1.4886096>

2.	J. A. Delgado-Notario, S. R. Power, W. Knap, et al., “Unveiling the Miniband Structure of Graphene Moiré Superlattices via Gate-Dependent Terahertz Photocurrent Spectroscopy”, *ACS Nano*, 19, 30 (2025). <https://doi.org/10.1021/acsnano.5c05306>

3.	I. K. Domaratskiy, M. A. Kashchenko, V. A. Semkin, et al., “Natural Edge Bilayer Graphene Transistor”. *Russian Microelectronics*, 52 (Suppl 1), S2–S5 (2023).. <https://doi.org/10.1134/S1063739723600541>

4.	S. Y. Chou, D. A. Antoniadis, H. I. Smith, “Application of the Shubnikov-de Haas oscillations in the characterization of Si MOSFET's and GaAs MODFET's”. *IEEE Trans. Electron Devices*, 34, 4, (1987). <https://doi.org/10.1109/T-ED.1987.23011>.

5.	J. M. Caridad, Ó. Castelló, S. M. López Baptista, et al., “Room-Temperature Plasmon-Assisted Resonant THz Detection in Single-Layer Graphene Transistors”, *Nano Letters*, 24, 3 (2024). <https://doi.org/10.1021/acs.nanolett.3c04300>

6.	D. A. Bandurin, D. Svintsov, I. Gayduchenko, et al., “Resonant terahertz detection using graphene plasmons”, *Nat. Commun.*, 9, 5392 (2018).

<https://doi.org/10.1038/s41467-018-07848-w>

7.	A. Satou, V. Ryzhii, V. Mitin, N. Vagidov “Damping of Plasma Waves in Two-Dimensional Electron Systems Due to Contacts”, *Physica status solidi (b)*, 246, 9 (2009). <https://doi.org/10.1002/PSSB.200945269>

8.	A. Shchepetov, C. Gard̀s, Y. Roelens, et al., “Oblique Modes Effect on Terahertz Plasma Wave Resonant Detection in InGaAs∕InAlAs Multichannel Transistors”. *Appl. Phys. Lett.*, 92, 242105 (2008). <https://doi.org/10.1063/1.2945286>

9.	D. Svintsov, “Hydrodynamic-to-Ballistic Crossover in Dirac Materials”, *Phys. Rev. B*, 97, 121405 (2018). <https://doi.org/10.1103/PhysRevB.97.121405>

10.	S. Boubanga-Tombet, F. Teppe, D. Coquillat, et al., “Current Driven Resonant Plasma Wave Detection of Terahertz Radiation: Toward the Dyakonov–Shur Instability”, Appl. Phys. Lett. 92, 212101 (2008). <https://doi.org/10.1063/1.2936077>
